# Cancer—Yesterday, Today, Tomorrow

**DOI:** 10.3390/medicina59010098

**Published:** 2022-12-31

**Authors:** Valentin Titus Grigorean, Daniel Alin Cristian

**Affiliations:** 1 “Bagdasar-Arseni” Clinical Emergency Hospital, 041915 Bucharest, Romania; 2 “Carol Davila” University of Medicine and Pharmacy, 050474 Bucharest, Romania; 3 “Colţea” Clinical Hospital, 030167 Bucharest, Romania

The COVID-19 pandemic has brought infectious and contagious diseases back to the forefront of medical concerns worldwide [1]. Prior to this period, the global mosaic of pathologies was dominated by cardiovascular diseases and cancers, as a cumulative result of the risk factors of our civilization [2,3].

Oncological pathology, unfortunately, has permanently occupied an undesirable leading position, even if, in previous decades, it was underdiagnosed. The more consistent application of screening methods and the improvement of diagnostic tools have given us an understanding that is closer to the true dimension of the phenomenon [4].

Genetic, molecular, histopathological, immunohistochemical, clinical and statistical studies have highlighted new facets of the neoplastic disease and found certain mutations suffered due to it (appearance at younger and younger ages, atypical evolutions, accompanying bizarre paraneoplastic phenomena, resistance to established therapeutic means, more frequent and more aggressive recurrence, etc.) [5,6].

The increase in average life expectancy has also induced an increase in the incidence of neoplasms in older people, with the appearance of new histological or evolutive types and the association of several concurrent or successive neoplasms in the same person [7,8].

Given that almost any component structure of the human body can develop multicellular malignant degeneration, with the possibility that the biology and “own strategy” of development and manifestation are different for each; that confirmed histological forms evolve (in the sense of worsening) from one stage to another; and that there is a loss of efficiency of some therapeutic means used (increasing resistance to chemotherapy, drug tachyphylaxis, radioresistance, etc.), we have a more complete picture of this puzzle [9,10].

The therapeutic means, in turn, have been developed and improved considerably, but must be permanently aligned with the dynamics of the development and manifestation of the neoplastic disease. Oncological surgery is often the pivot in cancer treatment as a practice of radical interventions on the primary tumor and resectable metastases. Unfortunately, it is possible to reach a capping of the surgical performance, considering that multivisceral resection (in locoregionally advanced forms) is not always applicable, and sometimes, its consequences are incompatible with survival or generate unacceptable suffering or sequelae. Often, oncological cases are completely inoperable and only palliative interventions can be practiced [11,12].

Transplantation surgery in oncology has obtained encouraging results, but currently, it is only applicable in a limited number of cancers that are in an early evolutive stage [13].

From this perspective emerges the idea that superior results in the fight against this disease can be obtained through a better understanding of predisposing genetic factors, of the particular neoplastic biology of each individual variant, of risk factors (environment, food, toxins, etc.) and of the development of nonsurgical therapeutic methods.

The COVID-19 pandemic has also produced profound changes regarding the issue of oncological pathology. It is worth noting, on the one hand, the reluctance of patients to access medical services during that period, and on the other hand, the overcrowding of medical units with the prioritization of medico-surgical emergencies.

Certainly, the effect of the SARS-CoV-2 infection on the patient’s oncological background could potentiate immunosuppression, along with other systemic effects, but, thus far, this has been insufficiently studied. Medical researchers are also called to clarify the general biological, disimmunity, vascular and individual reactivity mutations produced by the virus in the body of oncological patients [14].

Despite the significant progress made in the area of diagnosis and treatment of neoplastic disease, there remains a huge field to explore, considering the current unknowns and the alert dynamics under which cancer evolves. Oncology is the medical field that requires the mobilization of most medical specialties in order to outline the particular profile of each individual patient and optimize the necessary treatment [15].

The results of research from the various fields that deal with oncological issues must be capitalized and “assembled” into a unitary whole in order for them to have scientific and practical value.

This is the direction in which this Special Issue of the *Medicina* journal is headed, and is an invitation for all specialists working in the oncological field to present their experience and research results.
